# Draft Genome Sequence of the Moderately Heat-Tolerant *Lactococcus lactis* subsp. *lactis* bv. diacetylactis Strain GL2 from Algerian Dromedary Milk

**DOI:** 10.1128/genomeA.01334-15

**Published:** 2015-11-19

**Authors:** Noujoud Gabed, Manli Yang, Mohamed Bey Baba Hamed, Habiba Drici, Roy Gross, Thomas Dandekar, Chunguang Liang

**Affiliations:** a Department of Biotechnology, Université d'Oran 1 Ahmed Benbella, Oran, Algeria; b Department of Bioinformatics, Biocenter, University of Würzburg, Würzburg, Germany; c Department of Microbiology, Biocenter, University of Würzburg, Würzburg, Germany; d Biology Department/Laboratory of Sciences and Environment, Tamanghasset University Center, Tamanrasset, Algeria

## Abstract

*Lactococcus lactis* subsp. *lactis* bv. diacetylactis GL2 is a moderately thermotolerant lactic acid bacterium isolated from dromedary raw milk. Here, we present the draft genome sequence of this potential new dairy starter strain, which combines thermotolerance and the capacity to metabolize lactose, casein, and citrate.

## GENOME ANNOUNCEMENT

Dromedary milk constitutes an important protein source in pastoral societies of several countries; it is traditionally fermented by a natural flora that contains a panel of lactic acid bacteria, microorganisms which are widely used in food fermentations. A number of *Lactococcus* genome sequences have been determined (e.g., 1–3). In this work, we announce the draft genome sequence of the atypical *Lactococcus lactis* subsp. *lactis* bv. diacetylactis strain GL2, isolated from dromedary raw milk in south Algeria and formerly designated HD9B (4). In contrast to most other lactococci, this strain showed noticeable growth up to a temperature of 45°C. This thermotolerance probably reflects an adaptation to the high temperatures of the Algerian desert and is an interesting technological trait for cheese-making processes. In addition, the strain grows rapidly in cow’s milk and is able to ferment citrate, lactose, and many other carbohydrates, properties that have also been reported for *L. lactis* subsp. *lactis* and some bv. diacetylactis strains isolated from plant material (5).

*L. lactis* GL2 was cultured at 30°C in M17 medium (6) with 1% glucose. Total genomic DNA was isolated with the DNeasy blood and tissue kit (Qiagen). TruSeq DNA library preparations led to inserts of 250 to 650 bp. We collected 780-Mb paired-end (PE) raw reads from an Illumina MiSeq, and the quality was checked by FastQC (http://www.bioinformatics.babraham.ac.uk/projects/fastqc). FLASH (7) was applied for merging overlapping paired-end reads, and both adapters (p5 and p9) were clipped from these reads (merged and unconnected PE) using cutadapt (8). All low-quality tails (<25) were removed. *k*-mers were counted with Jellyfish (9), and the results were used to correct reads by the QUAKE tool (10). The assembly was carried out using Velvet (11) and IDBA-UD (12). Overlapping small contigs resulted in 48 contigs (>5 kb), totaling 2,245,404 bp, with an average G+C content of 35.1%.

Phylogenetic analysis using the maximum-likelihood method indicated *L. lactis* subsp. *lactis* KLDS4.0325 (3) isolated from kumis as the closest related sequenced strain, and the next closest was IL1403 (1). Genome functional annotation was performed with RAST 4 (13) and resulted in 2,203 protein-coding sequences (CDSs), among them a number of strain-specific CDSs, 43 tRNAs, and 4 rRNAs. The presence of chromosomal genes for diacetyl/acetoin production was confirmed. In addition, 39 noncoding RNAs were annotated by Infernal/Rfam (14).

A detailed analysis of the genome will give further insights into the genetic basis of the biotechnologically important traits of this strain.

### Nucleotide sequence accession numbers.

The whole-genome shotgun project has been deposited in GenBank/EMBL/DDBJ under the accession no. JNCC02000000 (chromosome), consisting of sequences JNCC02000001 to JNCC02000048.

## References

[1] Bolotin A, Wincker P, Mauger S, Jaillon O, Malarme K, Weissenbach J, Ehrlich SD, Sorokin A 2001 The complete genome sequence of the lactic acid bacterium *Lactococcus lactis* ssp. *lactis* IL1403. Genome Res 11:731–753. doi:10.1101/gr.GR-1697R. 11337471 PMC311110

[2] Falentin H, Naquin D, Loux V, Barloy-Hubler F, Loubière P, Nouaille S, Lavenier D, Le Bourgeois P, Francois P, Schrenzel J, Hernandez D, Even S, Le Loir Y 2014 Genome sequence of *Lactococcus lactis* subsp. *lactis* bv. diacetylactis LD61. Genome Announc 2(1):e01176-13. doi:10.1128/genomeA.01176-13. 24435871 PMC3894285

[3] Yang X, Wang Y, Huo G 2013 Complete genome sequence of *Lactococcus lactis* subsp. *lactis* KLDS4.0325. Genome Announc 1(6):e00962-13. doi:10.1128/genomeA.00962-13. 24285665 PMC3869327

[4] Drici H, Gilbert C, Kihal M, Atlan D 2010 Atypical citrate-fermenting *Lactococcus lactis* strains isolated from dromedary’s milk. J Appl Microbiol 108:647–657. doi:10.1111/j.1365-2672.2009.04459.x. 19663815

[5] Nomura M, Kobayashi M, Narita T, Kimoto-Nira H, Okamoto T 2006 Phenotypic and molecular characterization of *Lactococcus lactis* from milk and plants. J Appl Microbiol 101:396–405. doi:10.1111/j.1365-2672.2006.02949.x. 16882147

[6] Terzaghi B, Sandine W 1975 Improved medium for lactic *streptococci* and their bacteriophages. Appl Environ Microbiol 29:807–813. 10.1128/am.29.6.807-813.1975 PMC187084 16350018

[7] Magoč T, Salzberg SL 2011 FLASH: fast length adjustment of short reads to improve genome assemblies. Bioinformatics 27:2957–2963. doi:10.1093/bioinformatics/btr507. 21903629 PMC3198573

[8] Martin M 2011 Cutadapt removes adapter sequences from high-throughput sequencing reads. EMBnet.journal 17:10–12. doi:10.14806/ej.17.1.200.

[9] Marçais G, Kingsford C 2011 A fast, lock-free approach for efficient parallel counting of occurrences of *k*-mers. Bioinformatics 27:764–770. doi:10.1093/bioinformatics/btr011. 21217122 PMC3051319

[10] Kelley DR, Schatz MC, Salzberg SL 2010 Quake: quality-aware detection and correction of sequencing errors. Genome Biol 11:R116. 21114842 10.1186/gb-2010-11-11-r116 PMC3156955

[11] Zerbino DR, Birney E 2008 Velvet: algorithms for *de novo* short read assembly using de Bruijn graphs. Genome Res 18:821–829. doi:10.1101/gr.074492.107. 18349386 PMC2336801

[12] Peng Y, Leung HCM, Yiu SM, Chin FYL 2012 IDBA-UD: a *de novo* assembler for single-cell and metagenomic sequencing data with highly uneven depth. Bioinformatics 28:1420–1428. doi:10.1093/bioinformatics/bts174. 22495754

[13] Overbeek R, Olson R, Pusch GD, Olsen GJ, Davis JJ, Disz T, Edwards RA, Gerdes S, Parrello B, Shukla M, Vonstein V, Wattam AR, Xia F, Stevens R 2014 The SEED and the rapid annotation of microbial genomes using subsystems technology (RAST). Nucleic Acids Res 42:D206–D214. doi:10.1093/nar/gkt1226. 24293654 PMC3965101

[14] Nawrocki EP, Eddy SR 2013 Infernal 1.1: 100-fold faster RNA homology searches. Bioinformatics 29:2933–2935. doi:10.1093/bioinformatics/btt509. 24008419 PMC3810854

